# Chronic tophaceous gout

**DOI:** 10.11604/pamj.2021.40.130.31983

**Published:** 2021-11-02

**Authors:** Husam Bader, Rama Maghnam

**Affiliations:** 1 Monmouth Medical Center, Long Branch, New Jersey, United States of America

**Keywords:** Chronic, tophaceous, gout

## Image in medicine

Fifty-eight (58) year-old male with gouty arthritis for over 12 years, other medical history pertinent for hypertension, CKD stage 3 and heavy alcohol use. Patient described irregular usage of colchicine and urate-lowering drug for the last 10 years due to noncompliance with therapy. On physical examination, the patient was confined to a wheelchair because of his severe arthritis, he had deforming tophi and active synovitis of bilateral small joints of the hands (A), flexor tendon contractures (B), massive right big toe tophus (C) and concomitant septic arthritis and tophaceous gout of the left foot and ankle (D). Laboratory data were as follows: uric acid, 8.9 mg/dl; C-reactive protein (CRP): 33.5 mg/dl; creatinine, 2.8 mg/dl; blood and left ankle fluid cultures grew MRSA. Patient was treated emergently with IV antibiotics and subsequent below knee amputation as the foot was deemed clinically and radiographically unsalvageable. Patient was later started on febuxostat. After 4 months of therapy with febuxostat, he had no active synovitis. He was able to get out of the wheelchair and his CRP (0.4 mg/dl) and uric acid (4.2 mg/dl, the target is < 5 mg/dl) levels normalized (D). In addition, not only the tophaceous burden lessened but also the joint mobility improved, probably owing to control of the inflammation.

**Figure 1:**
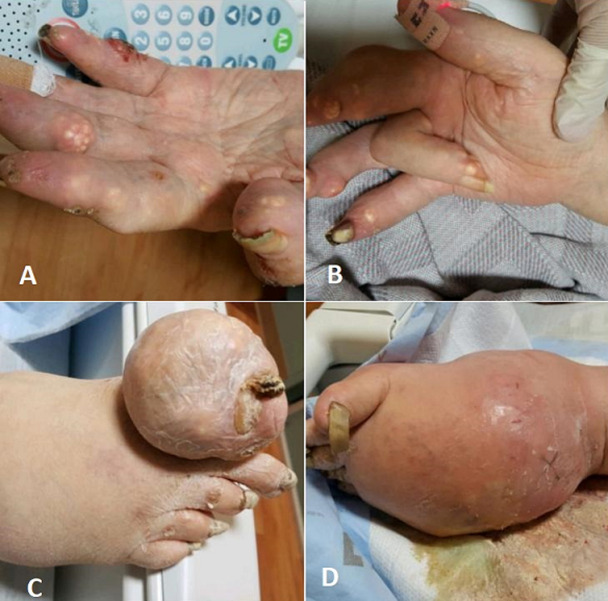
chronic tophaceous gout due to medication non-compliance; deforming tophi and active synovitis of bilateral small joints of the hands (A), flexor tendon contractures (B), massive right big toe tophus (C) and concomitant septic arthritis and tophaceous gout of the left foot and ankle (D)

